# Molecular Mechanisms of the Biological Control of Pine Wilt Disease Using Microorganisms

**DOI:** 10.3390/microorganisms13061215

**Published:** 2025-05-26

**Authors:** Xiaotian Su, Yimou Luo, Jingfei Hu, Yixin Xia, Min Liu, Yongxia Li, Haihua Wang

**Affiliations:** 1 College of Life Sciences, Shanxi Agricultural University, Jinzhong 030800, China; suxiaotian2023@163.com (X.S.); l15035907798@163.com (Y.L.); x3545069570@163.com (Y.X.); 2 College of Technology and Data, Yantai Nanshan University, Yantai 265706, China; wanghhstb@gmail.com; 3 College of Agriculture and Forestry, Linyi University, Linyi 276000, China; wangzhen@lyu.edu.cn; 4 Key Laboratory of Forest Protection of National Forestry and Grassland Administration, Ecology and Nature Conservation Institute, Chinese Academy of Forestry, Beijing 100091, China; 5 Department of Soil, Water, and Ecosystem Sciences, University of Florida, Gainesville, FL 32611, USA

**Keywords:** pine wilt disease, biocontrol microorganisms, nematicidal metabolites, vector insects, pine resistance

## Abstract

Pine wilt disease (PWD), caused by the pine wood nematode (PWN, *Bursaphelenchus xylophilus*), poses a significant threat to global pine forests and calls for the development of innovative management strategies. Microbial control emerges as an effective, cost-efficient, and environmentally sustainable approach to eliminate the damage from PWD. This review consolidates molecular mechanisms in the microbiological control of PWD, which focus on three core strategies: microbial control activity against PWN, biological control of vector insects, and the enhancement of host tree resistance to nematode infections. The review thoroughly evaluates integrated control strategies in which microbial control is used in traditional management practices. Recent studies have pinpointed promising microbial agents for PWN control, such as nematophagous microorganisms, nematicidal metabolites, parasitic fungi that target vector insects, and microbes that boost plant resistance. In particular, the control potential of volatile organic compounds (VOCs) produced by microorganisms against PWN and the enhancement of pine resistance to PWN by microorganisms were emphasized. Moreover, we assessed the challenges and opportunities associated with the field application of microbiological control agents. We emphasized the feasibility of multi-strategy microbial integrated control, which provides a framework for future studies on microbial-based PWD control strategies.

## 1. Introduction

**Figure 1:**
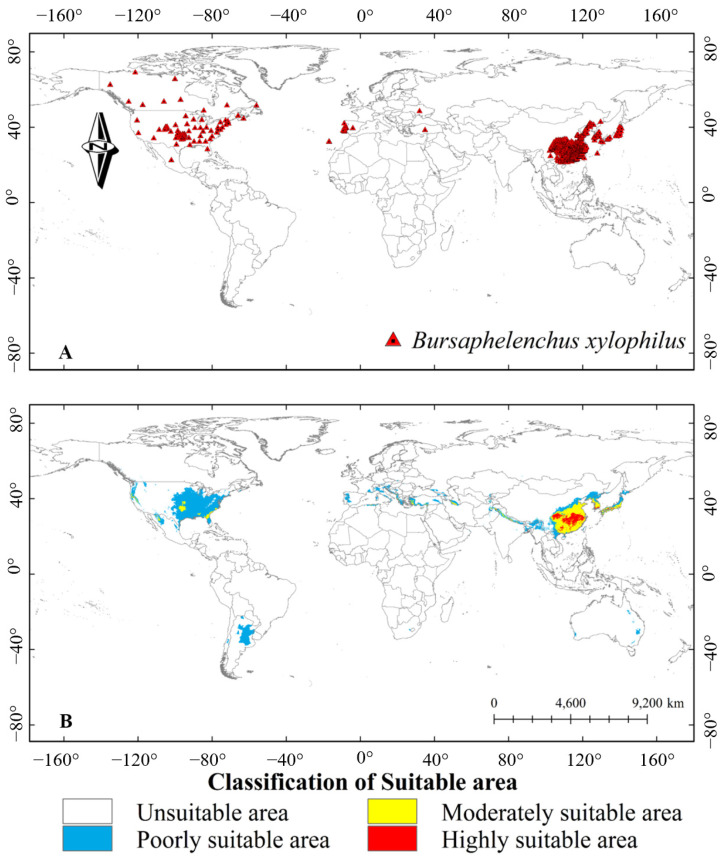
The global distribution and predicted distribution of pine wood nematode (PWN). (**A**) Global geographic distribution of the occurrence points of PWN; (**B**) Predicted distribution of PWN suitable areas under current climatic conditions. Note: In the horizontal direction, the negative value is the west longitude, and the positive value is the east longitude; In the vertical direction, negative values are south latitudes and positive values are north latitudes. Adapted from Xiao et al. (2023) [3].

Pine wilt disease (PWD) caused by the pine wood nematode (PWN), *Bursaphelenchus xylophilus*, which causes the rapid death of infected pine trees within a few weeks, has been a threat to pine forests worldwide (Figure 1) [1,2,3]. As a devastating disease to pine, PWN has invaded large-scale pine forests in China, Japan, Korea, and Europe, resulting in severe economic and ecological losses [4,5,6,7]. In East Asia, PWN first attacked Japan in 1905 and spread to China in 1982 [5,8,9,10]. A targeted monitoring of PWN based on the PWD risk assessment framework showed that such pine forest disease has been distributed in the southern, eastern, northeastern, northwestern, and central regions of China, with unsaturated geographical distribution and rapid spread [11]. Furthermore, global climate change accelerates in PWN distribution at high-latitude regions [12].

Currently, the control of PWD generally relies on a combination of physical and chemical measures. Physical measures, including fumigation of infected wood and black light trapping of vector insects, are highly expensive and labor-intensive [13]. The chemical agents, including avermectin, thiacloprid, milbemectin, alkaloids, terpenes, phenylpropanes, coumarins, and flavones [14,15], are rising concerns of serious environmental pollution [16]. Meanwhile, long-term application of chemical agents can induce resistance in PWN populations [17]. Biological control is an emerging approach that uses living organisms or their metabolites to reduce populations of PWN in pine forests [18], which is an emerging research hotspot [19,20]. Biological control measures, such as the application of insects like *Scleroderma guani*, have shown great effect in killing PWN [21,22]. Given their efficiency and environmental sustainability, the emerging microbial control measures demonstrate greater application potential due to their high diversity, rapid reproduction, and strong environmental adaptability [23,24].

The control of PWD using microorganisms primarily through three approaches: biological control of PWN populations, biological control of vector insects, and the enhancement of host tree resistance to nematode infections [24]. For instance, several biocontrol microorganisms showed high potential in the biological control of PWD due to their production of diverse nematicidal metabolites, which have been successfully developed into biological control agents (BCAs) [25]. Meanwhile, a crucial approach to PWN management involves controlling its primary vector, *Monochamus alternatus* (Coleoptera: Cerambycidae), which facilitates rapid PWN dissemination throughout pine forest ecosystems [26,27]. Compared to conventional biological control strategies, such as the release of natural enemies of *M. alternatus*, the application of entomopathogenic fungi, such as *Metarhizium* spp. and *Beauveria bassiana*, has emerged as an especially promising approach [22,28,29]. Furthermore, microorganism-mediated enhancement of pine resistance represents an emerging control strategy, operating through both constitutive improvement of host defenses and induced systemic resistance mechanisms. While the molecular and physiological mechanisms underlying these protective effects remain incompletely characterized, this approach holds considerable potential for integrated PWN management strategies.

To understand the molecular interactions between PWD and biological control microorganisms, we comprehensively reviewed the molecular mechanisms of microbial control of PWN through three fundamental aspects. First, we examined how microorganisms control the PWN population through two primary pathways: physical attack mechanisms and the production of nematicidal substances, with particular emphasis on volatile organic compounds (VOCs). Second, we summarized the interactions between microorganisms and PWN vectors, exploring how microbial agents can effectively control these vectors to prevent PWN transmission. Third, we evaluated how beneficial microorganisms can enhance pine tree resistance to PWN infection. We discussed these mechanisms in detail, with special attention to the molecular and biochemical pathways through which microorganisms induce enhanced plant resistance. Our work provides a new vision for developing an integrated biological control strategy against PWD.

## 2. Methodology

This study adopts the systematic literature review approach to investigate the molecular mechanisms of microbial-based control of PWD. The relevant keywords for literature search included “pine wilt disease”, “*Bursaphelenchus xylophilus*”, “pine wood nematode”, “biocontrol microorganisms”, “nematicidal metabolites”, “molecular mechanism”, “nematode-trapping fungi”, “endoparasitic fungi”, “toxigenic fungi”, “*Monochamus alternatus*”, “*Metarhizium*”, “*Beauveria*”, “vector insects”, “pine resistance”, “field application” alone and in combinations. The search engines included PubMed, Google Scholar, and CNKI. The search spanned from the establishment of each database up to 2024. Inclusion criteria were (1) the research clearly involves the biological control effects of microorganisms on PWN, vector insects, or pine trees; (2) provision of detailed molecular and biochemical mechanisms; (3) including experimental verification. Exclusion criteria were (1) literature with errors, incomplete data, or unavailable for analysis; (2) literature with inaccessible full text.

## 3. Molecular Mechanisms of Microbiological Control of PWN

A diverse array of microorganisms with potent nematophagous activity against PWN has been identified and characterized as promising biocontrol agents. These can be systematically classified into three major categories based on their control mechanisms: nematode-trapping fungi, which physically capture and kill nematodes; endoparasitic fungi, which parasitize nematodes; and toxigenic fungi, which produce compounds lethal to nematodes [24]. The specific microorganisms with demonstrated effects against PWN are listed in Table S1, providing a foundation for understanding potential biological control strategies.

### 3.1. Nematode-Trapping Microorganisms

Nematode-trapping fungi were isolated from pine-growing regions, such as Yunnan (China) and Choengdo City (Korea) [30,31]. These fungi are distinguished by their unique nematode-trapping structures, which mark their evolutionary transition from decomposers to predators. The trapping mechanisms can be categorized into two main types: constricting rings and adhesive structures (including adhesive knobs and three-dimensional adhesive networks). The fungal species, such as *Drechslerella dactyloides* and *Arthrobotrys dactyloides*, form constricting rings that effectively immobilize PWN [31,32]. Other fungi, including *Arthrobotrys cladodes* and *Arthrobotrys oviformis*, develop three-dimensional adhesive networks that bind to the nematode, facilitating subsequent infection and internal colonization [32]. Other fungi exhibiting these trapping capabilities include *Arthrobotrys*, *Dactylella*, and *Monacrosporium*. Recent research has demonstrated the effect of these fungi as biocontrol agents; for instance, *Volutella citrinella* GUCC2219 achieved a 33% predation rate in vitro, while its fermentation broth induced complete nematode mortality within 72 h [33].

The predation process of nematode-trapping fungi has been extensively studied. In the initial stages, these fungi produce VOCs, such as 2,4-dithiapentane, S-methyl thioacetate, and dimethyl disulfide, which mimic nematode pheromones to attract their prey [34]. Once attracted, the nematodes are recognized by the fungus *Arthrobotrys oligospora* through lectin–carbohydrate interactions mediated by lectins produced by the fungus [35]. Following recognition, the fungi initiate the formation of trapping structures to capture the nematodes. This process is regulated by in vivo signals [36]. Upon detecting nematodes, specific signaling pathways, including the G protein signaling pathway and Ca^2+^/calmodulin-dependent protein kinases (CaMKs), trigger the development of these structures [36]. The G protein pathway plays a critical role in trap formation in *A. oligospora*; for example, mutants lacking the G protein signal regulator *Ric8* are unable to form traps [37]. Similarly, CaMKs, a class of serine/threonine (Ser/Thr) kinases, contribute to various regulatory cascades within organisms [38]. In the *ΔCaMK B* mutant, where the *CaMK B* gene is knocked out, the formation of trapping structures is significantly delayed [39]. Once the fungi capture or adhere to the PWN, they secrete a suite of extracellular enzymes, including chitinase, serine protease Ac1, and collagenase. These enzymes act synergistically to degrade the nematode cuticle, facilitating fungal penetration and colonization [30,40,41]. Subsequently, *A. oligospora* penetrates the nematode via penetration tubes, exerting a mechanical force that causes the cuticle to indent and eventually breach [42]. In the final stage, the penetration tube develops into a nutrient mycelium at the infection site, which colonizes and digests the nematode, leading to its death [42,43].

The molecular studies suggested that the efficacy of nematode-trapping fungi relies on their secretion of various hydrolases, with serine protease identified as a particularly crucial enzyme in the trapping process [30,44]. A notable example is *Serratia* sp. A88copa13, isolated from Portugal, demonstrates high nematicidal activity through the production of a 70 kDa serine protease that actively degrades PWN components [44]. Similarly, *Arthrobotrys conoides* secretes an extracellular serine protease (Ac1) that immobilizes free-living PWN, showing 40–50% immobilization rates after 24 h of exposure [30].

### 3.2. Nematophagous Microorganisms

Nematophagous fungi represent a key group of microorganisms that control PWN through spore-based infection [45]. Unlike nematode-trapping fungi that rely on external capture mechanisms, endoparasitic fungi directly colonize the cavity of nematodes and result in rapid death, making them particularly effective as biocontrol agents. *Esteya vermicola*, an endoparasitic fungus demonstrating efficient infectivity against PWN [46]. This discovery marked a crucial advancement in biological control strategies against pine wilt disease, as *E. vermicola* exhibited both high specificity and pathogenicity toward PWN.

The infection mechanism of *E. vermicola* follows a sophisticated process. The fungus initially attracts PWN by producing specific volatile organic compounds (VOCs), including α-pinene, β-pinene, and camphor, which mimic pine tree scents [47]. These compounds were proved to be more attractive to PWN than those from actual pine trees [48]. Once PWN is attracted, *E. vermicola* initiates a four-day infection cycle [49]. The process begins when lunate conidia adhere to the cuticle of PWN without impeding its movement, enabling infected nematodes to disseminate the fungus throughout the host tree [50]. Within 18–24 h of adhesion, the conidia germinate and penetrate the cuticle of PWN [49] and then consume the internal contents of PWN, progressively reducing its motility and destroying its organs and tissues until death occurs [50]. The cycle concludes as fungal mycelium breaches the cuticle of a dead nematode, producing new lunate conidia that can attract and infect additional PWN in the next cycle [50].

Recent research has expanded our understanding of *Esteya* spp. and its infection mechanisms. Yin et al. (2020) demonstrated that *E. vermicola* blastospores exhibit comparable biocontrol efficacy to PWN [51]. Moreover, several strains of *E. vermicola* have been identified, including ATCC74485, CBS115803, CNU120806, and NKF13222. While *Esteya* spp. remained monotypic for many years after its discovery [46], Li et al. (2021) identified a second species, *Esteya floridanum*, which shares similar morphological characteristics and infection mechanisms with *E. vermicola*, demonstrating comparable nematicidal activity [52].

### 3.3. Toxigenic Microorganisms

Toxigenic microorganisms show significant potential in biocontrol against PWN due to their ability to produce nematicidal compounds, leading to rapid and effective nematode mortality. These organisms, including various species of fungi, bacteria, and actinomycetes, synthesize diverse classes of nematicidal metabolites, ranging from low-molecular-weight metabolites and volatile organic compounds (VOCs) to complex secondary metabolites and enzymes (Table 1). We specifically reviewed the toxigenic microorganisms identified to date and examined their specific nematicidal compounds, mechanisms of toxicity, and control efficiency against PWN, with particular emphasis on their potential for practical application in pine wilt disease management.

#### 3.3.1. Nematicidal Proteins from Toxigenic Microorganisms

Among toxigenic bacteria, the genus *Bacillus* has emerged as a source of nematicidal compounds against PWN, producing diverse bioactive molecules, including crystal proteins, guanidine compounds, and alkaline proteases [53,54,55]. *Bacillus thuringiensis* produces crystal protein App6Aa2, which demonstrates significant toxicity against PWN (LC50 = 49.71 μg·mL^−1^) by inducing shrinkage and thinning of intestinal cells, contraction of the intestine from the body wall, vacuolization, and the degeneration of appearance [53]. Similarly, *Bacillus* LYMC-3 synthesizes guanidine compounds with notable nematicidal activity, achieving LC50 values of 113.5 mg·L^−1^ and 62.5 mg·L^−1^ at 24 and 48 h, respectively [54]. Recent studies have shown that extracellular proteases from *Bacillus cereus* NJSZ-13 can degrade the nematode cuticle, progressively diminishing nematode activity [56]. Beyond natural isolates, genetic engineering approaches have yielded promising results, as demonstrated by the engineered strain Bxy19P3GFP, which expresses the Cry6Aa crystal protein and has shown significant nematicidal efficacy in greenhouse trials [57].

Recent studies have identified a novel nematotoxic cytolytic-like protein (CytCo) from the entomopathogenic fungus *Conidiobolus obscurus* that specifically targets the lipid metabolism of PWN through a distinct molecular mechanism. This protein disrupts the formation and maintenance of lipid droplets, which are crucial cellular organelles governing lipid homeostasis during PWN’s infection cycle. At the molecular level, CytCo operates through dual mechanisms: it interferes with surface phospholipid organization and modulates the expression of key genes involved in lipid metabolism. These combined effects lead to the elimination of large lipid droplets and subsequent disruption of lipid distribution patterns within PWN cells. While the immediate effects of CytCo on lipid metabolism are well-characterized, its broader implications for PWN’s infection cycle and potential applications in biocontrol strategies remain unclear [58]. Moreover, Actinomycetes, particularly *Streptomyces* species, produce diverse nematicidal compounds that show high toxicity against PWN. A prime example is teleocidin B4, isolated from *Streptomyces* sp. 680560, which achieves 95% mortality within 48 h while simultaneously inhibiting egg hatching of PWN [59]. The practical potential of these compounds is further evidenced by *Streptomyces* strain AE170020, whose extracts demonstrate complete PWN control at a minimal concentration of 7.2 mg per pine tree [60].

#### 3.3.2. Nematicidal Metabolites from Toxigenic Microorganisms

Toxigenic microorganisms synthesize diverse secondary metabolites that exhibit significant nematicidal activity against PWN. These nematicidal substances are classified into two primary categories based on their physicochemical properties: non-volatile metabolites and volatile organic compounds (VOCs). Previous investigations have led to the identification and characterization of 91 distinct nematicidal substances, comprising 54 non-volatile metabolites and 37 VOCs (Table 1). These compounds encompass multiple structural classes, including polyketones, lipopeptides, quinones, alkaloids, piperazines, phenols, terpenes, aldehydes, siderophores, and furans.

**Table 1:** Nematicidal metabolites against pine wood nematode secreted by microorganisms.

Strain	Substance Class	Bioactive Substance	Reference
Bacteria			
*Brevundimonas diminuta* LCB-3	Alcohols	(R)-(-)-2-ethylhexan-1-ol	[61]
*Serratia marcescens* AHPC29	Alkaloids	salsolinol	[59]
*Bacillus* sp. SMrs28	Alkenes	5,8-triene	[62]
*Streptomyces* sp. AN091965	Antibiotics	Spectinabilin	[63]
*Streptomyces ahygroscopicus*	Antibiotics	tetramycin B3	[64]
*Streptomyces* sp. AE170020	Aromatic Compounds	alloaureothin	[60]
*Streptomyces* sp. 680560	Aromatic Compounds	Teleocidin B4	[59]
*Bacillus* sp. SMrs28	Aromatic Compounds	phenylacetamide	[62]
*Lysinimonas M4*	Aromatic Compounds	2-coumaranone	[65]
*Streptomyces* sp. AE170020	Benzopyranones	Aureothin	[60]
*Bacillus* sp. SMrs28	Cyclic Compounds	4-Oxabicyclo [3.2.2] nona-1	[62]
*Stenotrophomonas maltophilia* G2	Enzymes	serine protease	[66]
*Bacillus* sp. SMrs28	Ester	methyl elaidate	[62]
*Bacillus* sp. SMrs28	Fatty Acids	lauric acid	[62]
*Bacillus* sp. SMrs28	Ketones	4-dione	[62]
*Streptomyces avermitilis* AVE-H39	Lactone	13α-Hydroxymilbemycinβ13	[67]
*Streptomyces avermitilis* AVE-H39	Lactone	26-methyl-13α-hydroxymilbemycin β13	[67]
*Bacillus pumilus* LYMC-3	Nitrogen Compounds	2-{3-[(3S,8aS)-1,4-dioxooctahydropyrrolo [1,2-a] pyrazin-3-yl] propyl} guanidine	[54]
*Bacillus* sp. SMrs28	Nitrogen Compounds	(3S, 8aS)-hexahydro-3methylpyrro [1,2-a] pyrazine-1	[62]
*Bacillus amyloliquefaciens* JK-JS3	Nitrogen Compounds	2,2-dimethyl-N-phenylpropanethioamide	[68]
*Bacillus amyloliquefaciens* JK-JS3	Nitrogen Compounds	Hexahydro-5-methyl-1-phenyl-1,3,5-triazine-2-thione	[68]
*Bacillus amyloliquefaciens* JK-JS3	Nitrogen Compounds	[(4,7,7-trimethyl-3-bicyclo [2.2.1] heptanylidene) amino] urea	[68]
*Streptomyces* sp. C611	Nitrogen Compounds	Furaltadone	[69]
*Bacillus* sp. SMrs28	Peptides	cyclo(L-Pro-L-Val)	[62]
*Lysinimonas M4*	Peptides	cyclo-(Phe-Pro)	[65]
*Erwinia* sp. A41C3	Siderophores	Catecholate-typesiderophore	[70]
*Rouxiella* sp. Arv20#4.1	Siderophores	hydroxamate-type siderophore	[70]
*Streptomyces* sp. TCS19-048	Sulfur compounds	S-3-1	[71]
*Pseudoduganella violaceinigra* G5-3	VOCs	2,5-dimethyl pyrazine	[72]
*Pseudoduganella violaceinigra* G5-3	VOCs	4-dimethylaminopyridine	[72]
*Pseudoduganella violaceinigra* G5-3	VOCs	benzyl acetate	[72]
*Pseudoduganella violaceinigra* G5-3	VOCs	phenethyl butyrate	[72]
*Pseudoduganella violaceinigra* G5-3	VOCs	phenethyl alcohol	[72]
*Stenotrophomonas maltophilia*	VOCs	phenol	[73]
*Bacillus subtilis*	VOCs	2-octanol	[73]
*Serratia marcescens*	VOCs	benzaldchyde	[73]
*Stenotrophomonas maltophilia*	VOCs	benzeneacetaldehyde	[73]
*Bacillus subtilis*	VOCs	decanal	[73]
*Bacillus subtilis*	VOCs	2-nonanone	[73]
*Stenotrophomonas maltophilia*	VOCs	2-undecanone	[73]
*Bacillus subtilis*	VOCs	cyclohexene	[73]
*Stenotrophomonas maltophilia*	VOCs	dimethyl disulfide	[73]
*Vibrio atlanticus* S-16 and *Pseudoalteromonas marina* H-42	VOCs	dimethyl disulfide	[74]
*Vibrio atlanticus* S-16 and *Pseudoalteromonas marina* H-42	VOCs	benzaldehyde	[74]
*Vibrio atlanticus* S-16 and *Pseudoalteromonas marina* H-42	VOCs	dimethyl trisulfide	[74]
*Vibrio atlanticus* S-16	VOCs	tert-butylamine	[74]
*Vibrio atlanticus* S-16	VOCs	acetone	[74]
*Pseudoalteromonas marina* H-42	VOCs	Dimethylamine	[74]
*Pseudoalteromonas marina* H-42	VOCs	N(diisopropylphosphino)methyl-	[74]
Fungi			
*Geotrichum* sp. AL4	Alcohols	[2,3-dihydro-2-(1-methylethenyl)-1-benzofuran-5-yl] methanol	[75]
*Alternaria* sp. Samif01	Aromatic Compounds	Alternariol 9-methyl ether	[76]
*Aspergillus fumigatus*	Aromatic Compounds	Fumiquinones A and B	[25]
*Caryospora callicarpa* YMF1.01026	Aromatic Compounds	4,8-Dihydroxy-3,4-dihydronaphthalen-1(2H)-one	[77]
*Caryospora callicarpa* YMF1.01026	Aromatic Compounds	4,6-dihydroxy-3,4-dihydronaphthalen-1(2H)-one	[77]
*Caryospora callicarpa* YMF1.01026	Aromatic Compounds	4,6,8-trihydroxy-3,4-dihydronaphthalen-1(2H)-one)	[77]
*Caryospora callicarpa* YMF1.01026	Aromatic Compounds	3,4,6,8-tetrahydroxy-3,4-dihydronaphthalen-1(2H)-one(cis-4-hydroxyscytalone)	[77]
*Oidiodendron* sp.	Aromatic Compounds	4-Hydroxyphenylacetic acid	[78]
*Gliocladium roseum* YMF1.00133	Aromatic Compounds	5-n-heneicosylresorcinol	[79]
*Geotrichum* sp. AL4	Aromatic Compounds	1-(2,4-dihydroxyphenyl) ethanone	[75]
*Caryospora callicarpa* YMF1.01026	Aromatic Compounds	caryospomycins A–C	[80,81]
*Coelomycetes* sp. YMFl.01029	Aromatic Compounds	Preussomerin C	[82]
*Coelomycetes* sp. YMFl.01029	Aromatic Compounds	preussomerin E	[82]
*Coelomycetes* sp. YMFl.01029	Aromatic Compounds	preussomerin D	[82,83]
*Coelomycetes* sp. YMFl.01029	Aromatic Compounds	4,6,8-trjhydfoxy-3,4-dihydronaphthalen-1(2H)-one	[82,83]
*Coelomycetes* sp. YMFl.01029	Aromatic Compounds	(4RS)4,8-dihydroxy-3,4-dihydronaphthalen-1(2H)-one	[82,83]
*Chaetomium ascotrichoides* 1-24-2	Aromatic Compounds	4,5,6-trihydroxy-7-methylphthalide	[84]
*Chaetomium ascotrichoides* 1-24-2	Aromatic Compounds	2-chlorobenzothiazole	[84]
*Fusarium oxysporum* EF119	Benzopyranones	Bikaverin	[85]
*Aspergillus* sp.	Carboxylic Acids	5-Hydroxymethyl-2-furoic acid	[86]
*Fusarium oxysporum* EF119	Carboxylic Acids	fusaric acid	[85]
*Pseudohalonectria adversaria* YMF1.01019	Cyclic Compounds	pseudohalonectrin A and B	[80]
*Fusarium bulbicola*	Cyclic Esters	Beauvericin	[87]
*Beauveria bassiana and Beauveria pseudobassiana*	Cyclic Esters	Beauvericin	[87]
*Paraniesslia* sp. YMF1.01400	Glycosides	(2S,2‘R,3R,3′E,4E,8E)-1-O-(β-D-glucopyranosyl)-3-hydroxyl-2-[N-2′-hydroxyl-3′-eicosadecenoyl] amino-9-methyl-4,8-octadecadiene	[80]
*Oidiodendron* sp.	Lactone	oidiolactone D	[78]
*Ophioceras dolichostomum* YMF1.00988	Lipids	Ophiocerol	[88]
*Geotrichum* sp. AL4	Nitrogen Compounds	1-[(2R*,4S*,5S*)-2-chloro-4-methyl-1,3-oxazinan-5-yl] ethenone	[75]
*Chaetomium ascotrichoides* 1-24-2	Nitrogen Compounds	*O*-methylisourea	[84]
*Gliocladium roseum* YMF1.00133	Peptides	Gliocladin C	[79]
*Gliocladium roseum* 1A	Peptides	Gliocladines A–D	[89]
*Trichoderma* sp.	VOCs	1β-vinylcyclopentane-1α,3α-diol	[83,90]
*Trichoderma* sp.	VOCs	6-pentyl-2H-pyran-2-one (2)	[83,90]
*Annulohypoxylon* sp. FPYF3050	VOCs	1,8-cineole	[91]
*Annulohypoxylon* sp. FPYF3050	VOCs	(+)-sativene	[91]
*Annulohypoxylon* sp. FPYF3050	VOCs	isocaryophyllene	[91]

The discovery of non-volatile nematicidal metabolites has advanced considerably since the initial characterization of aliphatic extracts from *Pseudohalonectria adversaria*, *Xylaria* sp., and *Hyphomycete* sp. exhibiting nematicidal activity against PWN [92]. Subsequent investigations led to the isolation and structural determination of increasingly potent compounds. Two naphthoquinone derivatives, Fumiquinones A and B, isolated from *Aspergillus fumigatus*, demonstrated nematicidal activities of 24% and 44%, respectively [25]. A significant advancement in this field was the identification of (R)-(-)-2-ethylhexan-1-ol from *Brevundimonas diminuta*, which exhibited 73% mortality against PWN in a 48 h bioassay [61]. Further structural diversity was revealed through the isolation of Beauvericin, a cyclohexadepsipeptide from *Fusarium bulbicola*, which demonstrated substantial nematicidal efficacy [93]. Recently, more efficient nematicidal substances have been identified, such as beauvericin, salsolinol, *O*-methylisourea, 2-chlorobenzothiazole, 4,5,6-trihydroxy-7-methylphthalide, and tetramycin B3 [64,84,87,94], showed high control efficiency against PWN.

VOCs have emerged as particularly efficacious nematicidal agents, consistently demonstrating promising nematicidal activity compared to non-volatile metabolites, with representative compounds including 2,5-dimethylpyrazine, 4-dimethylaminopyridine, and 1,8-cineole (Table 1). Quantitative bioassays have revealed the exceptional efficacy of bacterial VOCs, with metabolites from *Pseudoduganella violaceinigra* G5-3 and *Novosphingobium pokkalii* G8-2 achieving mortality rates of 98.26% and 93.10%, respectively, within 24 h of exposure [72]. Marine microorganisms are known to produce nematicidal VOCs, such as *Pseudoalteromonas marina* strain H-42 and *Vibrio atlanticus* strain S-16, which produce nematicidal compounds, including dimethyl trisulphide, benzaldehyde, dimethyl disulphide, and *tert*-butylamine [72]. Beyond direct nematicidal activity, certain VOCs exhibit developmental inhibition properties, as demonstrated by 1,8-cineole produced by *Annulohypoxylon* sp. FPYF3050, which significantly impairs PWN embryogenesis [91].

## 4. Molecular Mechanisms of Microbial Control of Vector Insects

*Monochamus alternatus* is the primary vector insect of PWN, spreading the nematode by feeding and laying eggs in healthy pine trees [95]. Several microorganisms, especially entomopathogenic fungi such as *Metarhizium* and *Beauveria* species, have shown significant potential as BCAs against *M. alternatus* [96]. This section examines the diverse microorganisms that show promise as BCAs and elucidates their infection mechanisms against *M. alternatus*.

### 4.1. Biological Control Agents of M. alternatus

Entomopathogenic fungi, particularly species of *Metarhizium* and *Beauveria*, have emerged as efficacious biological control agents against PWN due to their high virulence and demonstrated mortality rates (Table 2). Notably, *B. pseudobassiana* exhibited 100% mortality against *Monochamus galloprovincialis*, another PWN vector, at a concentration of 1 × 10^8^ conidia/mL [97]. Recent investigations by Zheng et al. (2024) revealed that *M. robertsii* GQH6, isolated from the Loess Plateau, achieved 100% corrected mortality against *M. alternatus* at concentrations of 10^8^ and 10^9^ conidia/mL [98]. Additionally, several other microorganisms have demonstrated promising potential as biological control agents against *M. alternatus* (Table 2). These include *Serratia marcescens* AHPC29, *S. nematodiphila* ZJPC33, *Lecanicillium decadeum*, *Aspergillus austwickii*, *Scopulariopsis alboflavescens*, *Aspergillus ruber*, *Beauveria bassiana*, *Penicillium citrinum*, and *Trichoderma dorotheae* [99,100]. These organisms have shown significant entomopathogenic activity while maintaining low phytotoxicity against host pine trees.

Climatic factors, such as temperature, humidity, and latitude, determine the distribution of *Metarhizium* and *Beauveria* and the pathogenicity effect on the *M. alternatus* [101]. Temperature has a significant impact on the efficacy of *Metarhizium* and *Beauveria* as BCAs [102]. Omuse et al. (2022) established a nonlinear model to simulate the effects of a wide temperature range on the conidial germination and mycelial growth of these fungi, identifying the optimal temperature for their pathogenicity and safety properties. Their results indicated that all fungi germinated best at 26.6–28.1 °C and grew best at 25.9–28.1 °C. These conditions are applicable to the management of most pests in tropical regions [103]. Therefore, establishing models to predict the adaptive growth temperatures of entomopathogenic fungi can help in selecting the most suitable fungi for different temperature regions. McGuire et al. (2022) proposed a qualitative conceptual model to study how latitude influences the specificity of fungi. In high-latitude regions, temperature is the most significant abiotic stressor, and fungi in these areas exhibit stronger environmental adaptability due to broader climate fluctuations. In contrast, fungi in low-latitude regions face greater selection pressure from host insects, making them more host-specific [104].

Under laboratory conditions, *B. bassiana* and *M. anisopliae* showed a great effect against *M. alternatus*. However, field conditions, influenced by environmental factors such as temperature and humidity, require evaluation of their stability in the control of PWN [105]. Notably, the *B*. *bassiana* ERL836 fungal powder demonstrated high nematicidal activity in the field. Compared to suspension sprays, which require large amounts of water, the fungal powder has a lower demand and is more applicable [106]. Furthermore, although Kim et al. (2022) suggest that the risk of *B*. *bassiana* ERL836-infected *M. alternatus* adults spreading the fungus to other non-target insects is low, the impact of *B. bassiana* and *M. anisopliae* on non-target insects and forest ecosystem security should still be taken seriously [106].

**Table 2:** Biocontrol microorganisms against vector insect, *Monochamus alternatus*.

Strain	Bioactive Substance	Killed Insects	Source	Reference
*B. bassiana*	N/A	*Monochamus alternatus*	*Monochamus alternatus*	[100,107]
*B. bassiana* F-263	N/A	*Monochamus alternatus*	*Monochamus alternatus*	[108,109,110]
*B. bassiana* ERL836	N/A	*Monochamus alternatus*	Entomology Research Laboratory, University of Vermont, USA	[106]
*B. bassiana* B7/B9	N/A	*Monochamus alternatus*	*Monochamus alternatus*	[111]
*B. brongniartii* F-877	N/A	*Monochamus alternatus*	*Monochamus alternatus*	[108]
*B. brongniartii* #879	N/A	*Monochamus alternatus*	*Psacothea hilaris*	[108]
*B. pseudobassiana*	N/A	*Monochamus galloprovincialis*	*Monochamus galloprovincialis*	[97]
*M. anisopliae* JEF-279	Destruxin and protease	*Monochamus alternatus*	Soil	[112]
*M. anisopliae* 1291	N/A	*Monochamus alternatus*	*M. alternatus* larva	[113]
*M. anisopliae* 1349	N/A	*Monochamus alternatus*	*M. alternatus* adult	[113]
*M. anisopliae* 2049	N/A	*Monochamus alternatus*	Cydnid bug adult	[113]
*M. anisopliae* JEF-197	N/A	*Monochamus alternatus*	Soil	[114]
*M. anisopliae* JEF-271	N/A	*Monochamus alternatus*	Soil	[114]
*M. anisopliae* JEF-279	N/A	*Monochamus alternatus*	Soil	[114]
*M. anisopliae* Ma789	N/A	*Monochamus alternatus*	Chinese Academy of Forestry	[115]
*M. anisopliae* MaYTTR-03	N/A	*Monochamus alternatus*	Soil	[116]
*M. anisopliae* MaYTTR-04	N/A	*Monochamus alternatus*	Soil	[116]
*M. anisopliae* MaZPTR-01	N/A	*Monochamus alternatus*	Soil	[116]
*M. anisopliae* var. *anisopliae*	N/A	*Monochamus alternatus*	*Monochamus alternatus*	[107]
*M. anisopliae* var. *major* CQMa117	N/A	*Monochamus alternatus*	*Monochamus alternatus*	[117]
*M. robertsii* GQH6	N/A	*Monochamus alternatus*	Soil	[98]
*Aspergillus austwickii*	N/A	*Monochamus alternatus*	*Monochamus alternatus*	[100]
*Aspergillus ruber*	N/A	*Monochamus alternatus*	*Monochamus alternatus*	[100]
*Bacillus thuringiensis* Cry3Aa	Coleopteran-specific Cry3Aa toxin	*Monochamus alternatus*	Not mentioned	[118]
*Lecanicillium attenuatum*	N/A	*Monochamus alternatus*	*Monochamus alternatus*	[100]
*Paecilomyces farinosus*	N/A	*Monochamus alternatus*	*Monochamus alternatus*	[107]
*Penicillium citrinum*	N/A	*Monochamus alternatus*	*Monochamus alternatus*	[100]
*Scopulariopsis alboflavescens*	N/A	*Monochamus alternatus*	*Monochamus alternatus*	[100]
*Serratia marcescens*	N/A	*Monochamus alternatus*	*Monochamus alternatus*	[107]
*Serratia marcescens* AHPC29	N/A	*M. alternatus* and *M. saltuarius*	*M. alternatus* and *M. saltuarius*	[99]
*Trichoderma dorotheae*	N/A	*Monochamus alternatus*	*Monochamus alternatus*	[100]

“N/A”: Not Applicable.

### 4.2. Infection Mechanisms of Metarhizium and Beauveria Against M. alternatus

The molecular mechanisms of the infection process of entomopathogenic fungi against *M. alternatus* have been well investigated and follow a complex, sequential pathway comprising six distinct stages: (1) conidial adhesion to the insect cuticle followed by germination; (2) enzymatic and mechanical penetration of the host cuticle; (3) proliferation of fungal hyphae within the insect cuticle; (4) production and secretion of insecticidal metabolites; (5) modulation of and response to host immune defenses; (6) fungal colonization culminating in hyphal extrusion and conidiation on the insect cadaver [119,120].

Initial colonization is mediated by specific fungal proteins that facilitate conidial attachment to the host cuticle. Fungal proteins, such as CFEM and Mad1, enable conidia to adhere to the host’s cuticle [121,122]. Mad1 also regulates cytoskeletal organization and activates genes related to the cell cycle [122]. Once attached, conidia produce germ tubes that develop into appressoria, structures enriched with organelles, preparing them to penetrate the host’s integument [123]. Cuticle penetration combines mechanical force and enzymatic breakdown [120]. Fungi like *Metarhizium* and *Beauveria* generate pressure through appressoria and secrete hydrolases, including proteases, chitinases, and lipases, to degrade the cuticle’s chitin, proteins, and lipids [119,124]. For instance, *M. anisopliae* employs the protease Pr1 to dismantle the cuticle of *M. alternatus* [125,126]. Other factors, such as MrGpa1 in *M. robertsii* and CYP52X1 in *B. bassiana*, further improve penetration efficiency [127,128].

Upon reaching the hemocoel, hyphae transform into yeast-like blastospores with a brush-like outer layer [129]. This adaptation helps the fungus evade the host’s immune defenses and spread rapidly within the hemolymph [119]. Fungi produce insecticidal metabolites, such as destruxins (DTXs) and proteases, which kill the host by inducing flaccid paralysis and disrupting the synthesis of nucleic acids and proteins [130,131]. Research by Kim et al. (2020) shows that DTXs and proteases from *M. anisopliae* strain JEF-279 cause flaccid paralysis in infected hosts [126]. In response, infected insects increase production of actin and tropomyosin, potentially as a defense against DTXs, with exposure to these metabolites leading to tetanic paralysis followed by flaccid paralysis [126]. Fungal infection prompts immune responses in *M. alternatus*, driven by the Toll and IMD pathways, which trigger antimicrobial peptide (AMP) production [132,133,134,135]. The reactive oxygen species (ROS) pathway also bolsters defense [132]. Additionally, bacteria such as *Pseudomonas* and *Serratia* associated with the insect inhibit *B. bassiana* by suppressing conidial germination and growth [136].

To counter these defenses, fungi employ strategies like immune evasion and enzyme secretion. For example, MCL1 in *M. anisopliae* conceals β-glucans on the fungal cell wall, reducing recognition by hemocytes [137]. The *MaAC* gene in the cAMP signaling pathway enhances fungal tolerance to oxidative and osmotic stress from the host’s immune system [138,139], while Ras GTPase (Ras3) and adenylate cyclase further support stress resistance [140,141]. When the host’s nutrients are exhausted, and death occurs, blastospores revert to hyphal growth and exit the cuticle, a process triggered by favorable environmental cues detected through G protein-mediated signaling [119]. The fungus then emerges, generates new conidia, and begins a new infection cycle [126].

## 5. Molecular Mechanisms of Microbial Enhancement of Pine Resistance Against PWN

Induced resistance (IR) is a phenomenon whereby plants, after appropriate stimulation, exhibit enhanced resistance to subsequent pathogen challenges [142]. This concept, first raised by Ross in the 1960s [143,144], primarily includes two distinct but interrelated defense mechanisms: systemic acquired resistance (SAR) and induced systemic resistance (ISR) [145]. SAR constitutes a defense mechanism activated through localized induction by virulent, avirulent, or non-pathogenic microbes, thereby enhancing the plant’s broad-spectrum defensive capacity against pathogens [145,146]. This process is characterized by elevated salicylic acid (SA) levels, a hormone central to immune responses against biotrophic pathogens [147], and concurrent activation of pathogenesis-related (PR) genes. These genes encode PR proteins with demonstrated antibacterial activity, which are functionally implicated in SAR-mediated defense [148]. In contrast, ISR is generally SA-independent and does not induce PR protein accumulation, distinguishing it from SAR [149]. ISR is primarily mediated by the phytohormones jasmonic acid (JA) and ethylene (ET) [145,150], functioning through enhancement of the host’s structural defenses rather than through direct pathogen killing or inhibition [146].

### 5.1. Mechanisms of Microorganisms Improve Pine SAR Against PWN

Pine trees exhibit enhanced resistance against PWN through several mechanisms regulated by microorganisms, phytohormones, secondary metabolites, oxidative stress responses, post-transcriptional regulation mediated by small RNAs, and the expression of defense-related genes [142,151]. The interaction between these factors contributes to the overall resilience of pine against pathogenic infections.

#### 5.1.1. Improvement of SAR by Exogenous Microorganisms

Exogenous microorganisms play a pivotal role in bolstering pine resistance against PWN. Upon PWN invasion, certain molecular mechanisms were applied by microorganisms to enhance pine resistance, especially by regulating the expression of defense genes in host pine, including encoding cell wall hydrolases and chitinases, or by inducing the synthesis of nematicidal compounds and enzymes, such as ethylene, peroxidase, catalase, and polyphenol oxidase [152,153,154]. Following PWN invasion, ethylene levels in pine trees tend to increase, indicating their potential role in the development of PWD [155]. However, excessive accumulation of the ethylene precursor, 1-aminocyclopropane-1-carboxylate (ACC), can lead to plant damage and even mortality [152,156]. Nascimento et al. (2013) demonstrated that ACC deaminase produced by *Pseudomonas putida* strain UW4 can mitigate the harmful ethylene levels in PWN-infected pine seedlings, thereby slowing down disease progression [152]. Furthermore, Nunes da Silva et al. (2019) indicated that a biofertilizer comprising diazotrophic bacteria and the chitosan-producing fungus *Cunninghamella elegans* prevented declines in photosynthetic pigments and water content in infected *Pinus pinaster*, while enhancing phenolic synthesis in PWN-inoculated *Pinus pinea* [157].

#### 5.1.2. Improvement of SAR by Endophytes

Endophytes associated with pine have demonstrated significant nematicidal activity and contribute to enhanced defensive capabilities against PWN [158]. For example, Peng et al. reported that the pine endophytic strain *Pseudomonas abietaniphila* BHJ04 significantly enhanced the growth of branches and roots of *Pinus massoniana* while inhibiting the spread of PWD [153]. Specifically, inoculation with strain BHJ04 resulted in a 64.97% increase in shoot dry weight and a 38.31% increase in root dry weight compared to control plants. Additionally, inoculation with strain BHJ04 led to a notable increase in the expression of cell wall hydrolase genes, chitinase genes, and genes belonging to the cytochrome P450 family in *P. massoniana*. Sun et al. (2024) identified the nematicidal properties of the endophyte *Bacillus velezensis* Pt-RP9, noting increased activities of peroxidase, catalase, and polyphenol oxidase in pine seedlings treated with this endophyte [154]. Given that pine trees typically engage in symbiotic relationships with various fungi, the impact of these microorganisms on PWN infection is notably significant [159,160,161,162]. For instance, while fungi such as *Aspergillus* can exacerbate PWN infections [163,164,165], beneficial biocontrol strains like *Curtobacterium pusillum* and *Pseudomonas putida* improve the resistance of pine trees against PWN [152,166].

#### 5.1.3. Improvement of SAR by Ectomycorrhizal Fungi

Ectomycorrhizal fungi (EMF) have also been implicated in enhancing resistance in pine trees against PWD. Nakashima et al. (2015) demonstrated that pine seedlings that formed highly abundant ectomycorrhizae displayed the highest survival and growth rates under PWN invasion, suggesting a beneficial role of EMF in conferring defense against PWN [167]. Notably, colonization rates of EMF in roots of PWN-infested trees were significantly reduced, indicating a potentially negative interaction between EMF colonization and PWN invasion [168]. Chu et al. (2019) identified various EMF species, including *Suillus lactifluus*, *Handkea utriformis*, *Amanita vaginata*, and *Suillus laricinus*, which enhanced host pine resistance against PWD [169]. Furthermore, since the pine rhizosphere microbiome is highly dependent on the growth of pine trees, PWN infection can severely damage the microbiome, leading to a decline in community diversity [149]. Chu et al. found that 9 months after inoculation with the ectomycorrhizal fungus *Suillus bovinus*, which significantly enhances pine resistance against PWN, the diversity and richness of the pine rhizosphere microbiome were significantly higher than in the control group [170]. Therefore, the long-term application of beneficial microorganisms may promote the growth of the pine rhizosphere microbiome by enhancing their resistance.

### 5.2. Mechanisms of Microorganisms Induce Pine ISR Against PWN

ISR is a plant-mediated resistance response primarily activated through ET and JA signaling pathways and is generally not associated with SA [171,172]. Certain rhizobacteria, like *Pseudomonas fluorescens* WCS417r, can trigger ISR without SA involvement [173], while others, such as *Pseudomonas aeruginosa* 7NSK2, may depend on SA induction, challenging the view that ISR is independent of SA [174]. Small RNAs are typically 20–30 nucleotide molecules, including microRNAs (miRNAs), small interfering RNAs (siRNAs), and piwi-interacting RNAs (piRNAs), which play important roles in regulating biological processes [175]. In plants, small RNAs can have both positive and negative effects on their resistance responses to pathogens [176]. Consequently, microorganisms can trigger ISR through two strategies. The first strategy involves promoting the expression of small RNAs that exert positive effects in plants. For instance, the expression of miR396d and miR408 is crucial for plant resistance responses [177,178]. When *B. subtilis* 26D triggers ISR in bread spring wheat against the aphid *Rhopalosiphum padi*, miR396d and miR408 are induced, and their expression levels increase sharply [179]. The second strategy is to inhibit the expression of small RNAs, which has negative effects on plants. For example, miR472 is a key regulator in *Arabidopsis* that negatively impacts plant disease-resistance genes. *Bacillus cereus* AR156 suppresses miR472 in *Arabidopsis*, thereby increasing the expression of the CC-NBS-LRR gene that miR472 targets, which subsequently triggers ISR in plants [180,181]. Similarly, *B. velezensis* FZB42 may trigger ISR by inhibiting the expression of zma-miR169a, zma-miR169c, and zma-miR169i. Understanding these multifaceted mechanisms enhances our ability to leverage beneficial microorganisms to improve plant resistance to PWN. Microbial induction of pine resistance to the PWN was first studied in Japan [182], applying an avirulent fungus, *Botrytis cinerea*, to PWN-infected black pine seedlings, demonstrating a decrease in average mortality from 97% to 89.9%, confirming that microorganisms can induce resistance in pine trees against PWN. One crucial defense response during pathogenic infections is the accumulation of pathogenesis-related proteins (PRs) [183]. Kim et al. (2019) discovered that genes associated with the PR-3 family (class I chitinase, class IV chitinase) and metallothionein-like proteins exhibited high transcript levels in calli treated with *Pseudomonas putida* 16YSM-E48, *Curtobacterium pusillum* 16YSM-P180, and *Stenotrophomonas rhizophila* 16YSM-M39 [184]. Additionally, Park et al. (2020) reported that *Bacillus thuringiensis* JCK-1233 could enhance PR expression in susceptible pine seedlings against PWN [166]. Han et al. (2021) illustrated the impact of microorganisms that induce resistance on the microbial diversity of pine trees. Treatment with *Pseudomonas koreensis* IRP7 and *Lysobacter enzymogenes* IRP8 led to increased relative abundance of beneficial microorganisms against PWN, including *Nitrospirillum*, *Bacillus*, *Luteibacter*, and obligate bacterial predators of the *Bdellovibrio* genus [185]. These findings underscore the potential of leveraging beneficial microorganisms to enhance resistance in pine trees against PWN.

## 6. The Application of Microbial BCAs

The field application of microbial control agents (BCAs) for managing the pine wood nematode (PWN) encounters several challenges, including reduced efficacy, elevated ecological safety risks, and high costs [186]. These obstacles are elaborated as follows: (1) Significant disparities exist between laboratory and field conditions for microbial BCAs. The broad latitudinal distribution of PWN demands that BCAs exhibit robust environmental adaptability [11]. Moreover, resistance in PWN and its vector insects, potentially mediated by multidrug resistance-associated protein (MRP) genes, can diminish BCA efficacy [17]. Application methods and conditions further influence performance. For example, a field test using *B. bassiana* ERL836 revealed varying mortality rates in *M. alternatus* depending on whether a spray suspension or fungal powder was used, as well as the concentration applied [106]. Consequently, comprehensive efficacy assessments are essential prior to BCA deployment to ensure optimal outcomes. (2) Environmental safety is a critical factor in microbial control strategies targeting PWN. Prolonged use of BCAs may affect local ecosystems, while some agents, such as *B. bassiana* ERL836, demonstrate minimal non-target effects [106]. Ecological risks persist due to diverse geographical conditions and forest types. Additionally, pine tree growth is closely tied to rhizosphere microorganisms. BCAs may help restore pine rhizosphere microbial communities [170]; however, a broader evaluation of their environmental impact is still required. Furthermore, certain BCAs are pathogenic, introducing biosafety concerns during production, storage, and transportation. (3) High costs represent a significant barrier to the widespread adoption of BCAs. Reducing production expenses, which often exceed those of traditional control agents, is imperative. For instance, solid-state fermentation (SSF) with rice as a carrier to produce *Esteya vermicola* conidia offers a scalable, cost-effective, and practical alternative to liquid fermentation [187]. Therefore, we propose that microbial consortia for field deployment meet the following standards: (1) high nematicidal activity; (2) low resistance risk; (3) strong environmental adaptability; (4) low ecological risk or pathogenicity after assessment; (5) low production cost in the field practice.

## 7. Conclusions

This review synthesizes the molecular mechanisms underpinning microbial control strategies against PWD caused by *Bursaphelenchus xylophilus*. This review makes three significant contributions to pine wilt disease management: (1) we systematically evaluate three prevention pathways through comparative mechanistic analysis, proposing synergistic integration strategies to enhance nematode control; (2) we identify key knowledge gaps in microbial control of PWN, emphasizing the need to investigate microorganism-induced systemic resistance, evaluate ecological impacts of microbial applications, and model climate-mediated pathogen-vector-microbe interactions; (3) we highlight bioinformatics and multi-omics integration as critical tools for elucidating biocontrol mechanisms while outlining field application challenges for microbial BCAs and establishing selection criteria for their practical deployment.

**Figure 2:**
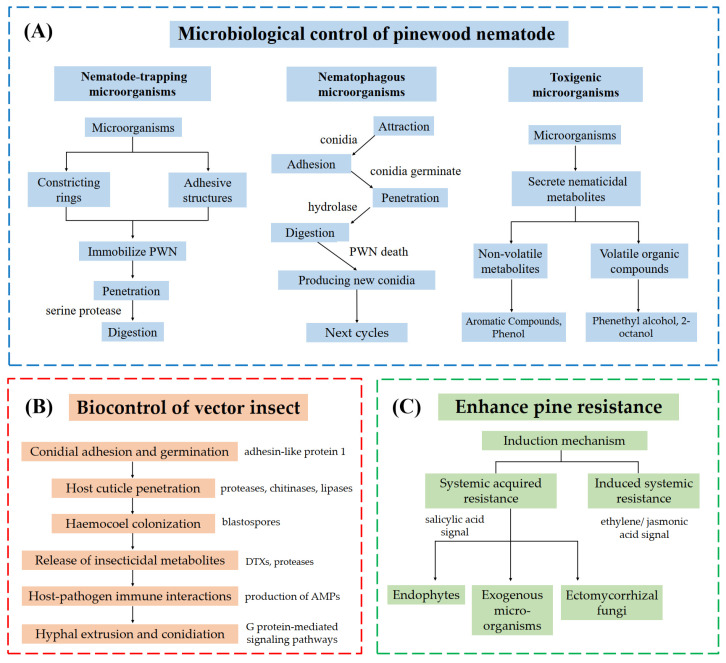
Three strategies for biological control of pine wilt disease. (**A**) Direct microbiological control employs three mechanisms: nematode-trapping microorganisms immobilize pine wood nematode via constricting rings or adhesive structures, followed by tissue digestion through hydrolases (e.g., serine proteases); nematophagous fungi adhere to the nematode cuticle via conidia, which germinate, penetrate using hydrolases (proteases, chitinases), and digest internal tissues, with new conidia produced post-mortem to propagate infection; toxigenic microorganisms secrete nematicidal metabolites, including volatile organic compounds (VOCs) and non-volatile toxins. (**B**) Biological control of insect vectors involves six stages: adhesin-like protein 1 mediates conidial adhesion and germination on the host cuticle; hydrolases (proteases, chitinases, lipases) degrade the cuticle for fungal penetration; blastospores colonize the hemocoel; insecticidal metabolites (e.g., destruxins [DTXs], proteases) suppress host immunity; antimicrobial peptides (AMPs) modulate host-pathogen immune interactions; G protein-mediated signaling induces hyphal extrusion and conidiation to initiate new infection cycles. (**C**) Pine resistance is enhanced via systemic defense priming: systemic acquired resistance (SAR, salicylic acid-mediated) and induced systemic resistance (ISR, ethylene/jasmonic acid-mediated).

The mechanisms of the microbiological control of PWD are supported by molecular insights into hydrolase activity, toxin biosynthesis, and plant defense gene regulation, offering a foundation for targeted biocontrol applications (Figure 2). The integration of bioinformatic tools and multi-omic techniques has revolutionized the study of molecular mechanisms in PWD biocontrol. Genomic sequencing of nematophagous fungi (e.g., *E. vermicola*) and bacteria (e.g., *B. thuringiensis*) has identified key nematicidal genes, such as those encoding proteases and Cry toxins [187,188,189], while transcriptomic analyses have unveiled host-pathogen interaction dynamics, including pine defense gene activation during induced systemic resistance [190,191,192]. Metabolomics has enabled the profiling of nematicidal compounds, such as VOCs from *P. violaceinigra*, linking chemical diversity to biocontrol efficacy [72]. Proteomic studies further elucidate enzymatic pathways critical for fungal penetration of nematode cuticles or insect exoskeletons [193]. Additionally, metagenomics provides insights into microbial community shifts in PWN-infected pines, guiding the design of ecologically compatible consortia [160,194]. Despite these advances, challenges remain in data harmonization and functional validation of omics-derived hypotheses, necessitating interdisciplinary collaboration to translate molecular discoveries into field applications.

An integrated approach combining microbial agents with traditional management practices holds significant potential for sustainable PWD control. For instance, pairing nematophagous fungi with pheromone-based vector trapping or leveraging VOCs for dual nematode suppression and pine priming could enhance efficacy. The synergistic interactions among different biocontrol microorganisms require further study. The toxigenic bacterium *Stenotrophomonas maltophilia*, which produces nematocidal VOCs, can induce trapping structure formation in the nematode-trapping fungus *A. oligospora* [195]. A recent study showed *S. maltophilia* also directly triggers trapping structures by endogenizing within and participating in the nitrogen cycle of *Arthrobotrys musiformis* [196]. Genetic engineering of microbial strains to overexpress nematicidal proteins (e.g., Cry6Aa in *B. toyonensis*) or optimize VOC production may further improve field performance. However, ecological compatibility must be prioritized to avoid disrupting native microbial communities. Regional PWN distribution patterns, as highlighted in Figure 1, necessitate tailored strategies, such as prioritizing vector control in newly invaded areas and resistance enhancement in endemic zones.

Critical research gaps persist in understanding molecular mechanisms. First, the precise pathways by which microbes induce SAR/ISR in pines—particularly the roles of phytohormones, small RNAs, and secondary metabolites—remain poorly characterized. Second, the ecological interplay between introduced biocontrol agents, native microbiota, and PWN in forest ecosystems is underexplored, raising concerns about long-term sustainability. Third, while lab studies demonstrate the high efficacy of microbial agents (e.g., *Pseudoduganella violaceinigra* VOCs achieving >90% nematode mortality), field applications often face challenges due to environmental variability and host-pathogen co-evolution. Fourth, it is crucial to study the molecular mechanisms by which different types of biocontrol microorganisms (e.g., nematode-trapping fungi and VOC-producing bacteria) exert synergistic effects. Additionally, the molecular basis of PWN resistance to microbial toxins and the impact of climate change on microbial-nematode-vector tripartite interactions require deeper investigation. Addressing these gaps will be pivotal for advancing biocontrol strategies that are both effective and ecologically resilient.

In conclusion, the integration of molecular insights, ecological considerations, and innovative technologies will drive the development of next-generation biocontrol solutions. Future research should prioritize elucidating unresolved molecular interactions, optimizing microbial consortia for field stability, and validating integrated strategies under diverse environmental conditions to safeguard global pine forests.

## Data Availability

No new data were created or analyzed in this study.

## Supplementary Materials

The following supporting information can be downloaded at: https://www.mdpi.com/article/10.3390/microorganisms13061215/s1, Table S1: Diversity of biocontrol microorganisms against PWN. References [197,198,199,200,201,202,203,204,205,206,207,208,209,210,211,212,213] are cited in the supplementary materials.
