# Supplementary material for: Molecular Mechanisms of the Biological Control of Pine Wilt Disease Using Microorganisms
Source: Microorganisms. 2025 May 26;13(6):1215. doi: 10.3390/microorganisms13061215 (PMC12195133; doi:10.3390/microorganisms13061215)
Supplement: Supplementary file 1 [file microorganisms-13-01215-s001.zip › microorganisms-3583440-supplementary.pdf]

Supplementary Table S1. Diversity of biocontrol microorganisms against PWN.

Strain	Category	Isolate Area	Source	Reference
**Fungi**				
Arthrobotrys conoides	Nematode-trapping	Yunnan Province, China	field soil sample	[30]
Arthrobotrys dendroides	Nematode-trapping	Ninghai City, Zhejiang province, China	diseased tree of black pine wilt	[197]
Arthrobotrys musiformis	Nematode-trapping	Ninghai City, Zhejiang province, China	diseased tree of black pine wilt	[197]
Arthrobotrys cladodes	Nematode-trapping	Fushun City, Liaoning Province, China	the xylem of dead and diseased *Pinus koraiensis*	[198]
Arthrobotrys cladodesr	Nematode-trapping	Ninghai City, Zhejiang province, China	diseased tree of black pine wilt	[197]
Arthrobotrys oligospora	Nematode-trapping	Ninghai City, Zhejiang province, China	diseased tree of black pine wilt	[197]
Arthrobotrys robusta	Nematode-trapping	Harbin City, Heilongjiang Province, China	soil	[198]
Arthrobotrys sp.	Nematode-trapping	Tsukuba, Ibaraki prefecture	pine sap	[199]
Arthrobotrys superba	Nematode-trapping	Wisconsin	red pines	[200]
Dactylellina ellipsospora	Nematode-trapping	Ninghai City, Zhejiang province, China	diseased tree of black pine wilt	[197]
Dactylellina dactyloides CNU 091025 and CNU 091026	Nematode-trapping	Choengdo, Korea	forest soil samples	[31]
Monacrosporium megalosporum	Nematode-trapping	Japan	soil and plant	[210]
Monacrosporium thaumasium	Nematode-trapping	Ninghai City, Zhejiang province, China	diseased tree of black pine wilt	[197]
Esteya floridanum	Endoparasitic	Austin Cary Forest, Gainesville, Florida	head of a male adult of *Myoplatypus flavicornis* on loblolly pine	[52]
Esteya vermicola	Endoparasitic	Yangmingshan, Taipei City, Taiwan	pinewood nematode-infected and wilted Japanese black pine trees with an increment borer	[46]

Esteya vermicola	Endoparasitic	central Bohemia, Czech Republic	Oak branches infested with Scolytus intricatus	[202]
Esteya vermicola CNU 120806	Endoparasitic	Choengdo, Korea	infected nematodes in forest soil samples	[203]
Esteya vermicola NKF 13222	Endoparasitic	wood packaging materials originating in Brazil and arriving at Tianjin port in China	an isolate of Bursaphelenchus rainulfi which was intercepted	[204]
Acremonium sp. BH0531	Toxigenic	Bohai-Bay (E117°37′10.6″–117°59′36.4″, N38°37′03.6″–38°55′51″) Tianjin, China	seawater	[205]
Aspergillus sp.	Toxigenic	Kitakyushu	soil	[86]
Camposporium quercicola YMF1.01300	Toxigenic	freshwater habitat in Yunnan Province, China	submerged woody substrate	[77]
Caryospora callicarpa YMF1.01026	Toxigenic	freshwater habitat in Yunnan Province, China	submerged woody substrate	[77]
Fusarium bulbicola	Toxigenic	Kitakyushu	soil	[93]
Geotrichum sp. AL4	Toxigenic	Xishuangbanna, Yunnan Province, P. R. China	leaves of the neem tree (Azadirachta indica)	[75]
Gliocladium roseum YMF1.00133	Toxigenic	fresh water in Yunnan Province, China	submerged wood	[79]
Melanospora zamiae YMF1.00948	Toxigenic	freshwater habitat in Yunnan Province, China	submerged woody substrate	[77]
Oidiodendron sp.	Toxigenic	Tottori	soil	[78]
Periconia digitata YMF1.00948	Toxigenic	freshwater habitat in Yunnan Province, China	submerged woody substrate	[77]
Pseudohalonectria adversaria YMF1.01019	Toxigenic	Yunnan Province, China	submerged wood	[206]
Trichoderma sp.	Toxigenic	Yunnan Province, China	soil	[90]
Xylaria sp.	Toxigenic	Guangzhou, China	the leaves, stems, and roots of *Quisqualis indica*	[207]
**Bacteria**				
Novosphingobium pokkalii G8-2	Toxigenic	Qingdao University campus	water, soil and biological samples	[72]
Pseudoduganella violaceinigra G5-3	Toxigenic	Qingdao University campus	water, soil and biological samples	[72]
Bacillus cereus NJSZ-13	Toxigenic	Nanjing Zhongshan Botanical Garden	healthy *Pinus elliottii* tree	[56]
Bacillus sp. EB 93	Toxigenic	Korea	pine tree	[157]

Bacillus sp. SMrs28	Toxigenic	Lanzhou, China	rhizosphere soil of *S. chamaejasme*	[62]
Bacillus thuringiensis	Toxigenic	Zhejiang, China	forests	[55]
Brevundimonas diminuta LCB-3	Toxigenic	Kunming Institute of Botany P. R. China	plant samples	[61]
Coelomycetes sp. YMFl.01029	Toxigenic	Lake Fuxian in Yunnan Province, China	split of decaying branches of an unidentified tree	[82]
Erwinia sp. A41C3	Toxigenic	Portugal	pine tree	[208]
Escherichia coli M131/M132	Toxigenic	Korea	pine tree	[209]
Lysinimonas sp. M4	Toxigenic	campus of Qingdao University	roots of *Pinus thunbergii*	[65]
Ophioceras dolichostomum YMF1.00988	Toxigenic	freshwater habitat in Yunnan Province, China	submerged woody substrate	[88]
Serratia marcescens M44	Toxigenic	Korea	pine tree	[209]
Stenotrophomonas sp. EB394	Toxigenic	Korea	pine tree	[157]
Streptomyces sp. 680560	Toxigenic	Korea	pine tree	[59]
Streptomyces sp. AE170020	Toxigenic	Jinju, Korea	root tissues of pine tree samples	[59]
Streptomyces sp.AN091965	Toxigenic	Korea	forest soil samples	[63]
Streptomyces termitum HT-8	Toxigenic	Qingdao, China	sea sand	[210]
Aspergillus fumigatus	Toxigenic	Kitakyushu	soil	[25]
Bacillus cereus JK-XZ3	Toxigenic	Xuzhou, China	rhizosphere soil of cherry blossom	[211]
Bacillus pumilus YH-20	Toxigenic	Shanghai, China	the stem of cherry	[211]
Bacillus velezensis HR10	Toxigenic	China	*P. thunbergii* and the mycorrhizal soil of *Gastrosporium simplex*	[211]
Bacillus thuringiensis 020	Toxigenic	no mentioned	National Key Laboratory of Agricultural Microblology	[212]
Bacillus thuringiensis RBT-200701	Toxigenic	no mentioned	Russian State Center for Applied Microbiology Research	[212]
Bacillus toyonensis Bxy19GFP	Toxigenic	Hangzhou, China	the xylem of pine	[57]

Enterobacter ludwigii AA4	Toxigenic	no mentioned	the root of maize	[213]
B. pumilus LYMC-3	Toxigenic	no mentioned	the stem of *Pinus massoniana*	[54]

References

30. Yang, J.; Li, J.; Liang, L.; Tian, B.; Zhang, Y.; Cheng, C.; Zhang, K.Q. Cloning and characterization of an extracellular serine protease from the nematode-trapping fungus *Arthrobotrys conoides*. *Arch. Microbiol.* **2007**, *188*, 167-174. https://doi.org/10.1007/s00203-007-0233-x

197. Zhang, H.; Wei, Z.; Zhang, J.; Liu, X. Classification of dendrocola nematode-trapping fungi. *J. For. Res.* **2021**, *32*, 1295-1304. https://doi.org/10.1007/s11676-020-01159-x

198. Jia, H.; Xia, R.; Zhang, R.; Liang, G.; Zhuang, Y.; Zhou, Y.; Li, D.; Wang, F. Transcriptome analysis highlights the influence of temperature on hydrolase and traps in nematode-trapping fungi. *Front. Microbiol.* **2024**, *15*, 1384459. https://doi.org/10.3389/fmicb.2024.1384459

199 Saiki, H.; Saito, T.; Yoneda, K.; Umi, M.; Uchida, K.; Yamanaka, K. Biological control of the pine-wood nematode by spraying a nematode-trapping fungus. *J. Jpn. For. Soc.* **1984**, *66*, 30-32. https://doi.org/10.1006/bcon.1995.1075

200. Wingfield, M.J. Fungi associated with the pine wood nematode, *Bursaphelenchus xylophilus*, and cerambycid beetles in Wisconsin. *Mycologia* **1987**, *79*, 325-328. https://doi.org/10.1080/00275514.1987.12025713

31. Wang, Z.; Wang, C.Y.; Gu, L.J.; Sun, B.S.; Zhang, D.L.; Liu, L.; Lee, M.R.; Wang, C.L.; Li, Z.; Mo, E.K.; et al. Variabilities of two *Drechslerella dactyloides* isolates in Korea and high predacity against *Bursaphelenchus xylophilus*. *Curr. Microbiol.* **2011**, *62*, 472-478. https://doi.org/10.1007/s00284-010-9731-1

201. Kano, S.; Aimi, T.; Masumoto, S.; Kitamoto, Y.; Morinaga, T. Physiology and molecular characteristics of a pine wilt nematode-trapping fungus, *Monacrosporium megalosporum*. *Curr. Microbiol.* **2004**, *49*, 158-164. https://doi.org/10.1007/s00284-004-4268-9

52. Li, Y.; Yu, H.; Araújo, J.P.M.; Zhang, X.; Ji, Y.; Hulcr, J. *Esteya floridanum* sp. nov.: An Ophiostomatalean nematophagous fungus and its potential to control the pine wood nematode. *Phytopathology* **2021**, *111*, 304-311. https://doi.org/10.1094/phyto-06-20-0229-r

46. Liou, J.; Shih, J.; Tzean, S. *Esteya*, a new nematophagous genus from Taiwan, attacking the pinewood nematode (*Bursaphelenchus xylophilus*). *Mycol. Res.* **1999**, *103*, 242-248. https://doi.org/10.1017/s0953756298006984

202. Kubátová, A.; Novotný, D.; Práil, K.; Mrácek, Z. The nematophagous hyphomycete *Esteya vermicola* found in the Czech Republic. *Czech Mycol.* **2000**, *52*, 227-235. https://doi.org/10.33585/cmy.52305

203. Wang, C.Y.; Fang, Z.M.; Sun, B.S.; Gu, L.J.; Zhang, K.Q.; Sung, C.K. High infectivity of an endoparasitic fungus strain, *Esteya vermicola*, against nematodes. *J. Microbiol.* **2008**, *46*, 380-389. https://doi.org/10.1007/s12275-007-0122-7

204. Wang, X.; Wang, T.; Wang, J.; Guan, T.; Li, H. Morphological, molecular and biological characterization of *Esteya vermicola*, a nematophagous fungus isolated from intercepted wood packing materials exported from Brazil. *Mycoscience* **2014**, *55*, 367-377. https://doi.org/10.1016/j.myc.2014.01.002

205. Meng, Q.; Shi, X.; Meng, F.; Feng, X.; Sun, J. Isolation of an *Acremonium* sp. from a screening of 52 seawater fungal isolates and preliminary characterization of its growth conditions and nematicidal activity. *Biotechnol. Lett.* **2012**, *34*, 1847-1850. https://doi.org/10.1007/s10529-012-0967-7

86. Kimura, Y.; Tani, S.; Hayashi, A.; Ohtani, K.; Fujioka, S.; Kawano, T.; Shimada, A. Nematicidal activity of 5-hydroxymethyl-2-furoic acid against plant-parasitic nematodes. *Z Naturforsch C.* **2007**, *62*, 234-238. https://doi.org/10.1515/znc-2007-3-413

77. Zhu, Y.; Dong, J.; Wang, L.; Zhou, W.; Li, L.; He, H.; Liu, H.; Zhang, K. Screening and isolation of antinematodal metabolites against *Bursaphelenchus xylophilus* produced by fungi. *Ann. Microbiol.* **2008**, *58*, 375-380. https://doi.org/10.1007/bf03175531

93. Shimada, A.; Fujioka, S.; Koshino, H.; Kimura, Y. Nematicidal activity of beauvericin produced by the fungus *Fusarium bulbicola*. *Z Naturforsch C.* **2010**, *65*, 207-210. https://doi.org/10.1515/znc-2010-3-407

75. Li, G.H.; Yu, Z.F.; Li, X.; Wang, X.B.; Zheng, L.J.; Zhang, K.Q. Nematicidal metabolites produced by the endophytic fungus *Geotrichum* sp. AL4. *Chem. Biodivers.* **2007**, *4*, 1520-1524. https://doi.org/10.1002/cbdv.200790131

79 Song, H.; Shen, W.; Dong, J. Nematicidal metabolites from *Gliocladium roseum* YMF1. 00133. *Appl. Biochem. Microbiol.* **2016**, *52*, 324-330. https://doi.org/10.1134/s0003683816030169

78. Ohtani, K.; Fujioka, S.; Shimada, A.; Kimura, Y. Nematicidal activities of 4-hydroxyphenylacetic acid and oidiolactone D produced by the fungus *Oidiodendron* sp. *Z Naturforsch C.* **2011**, *66*, 31-34. https://doi.org/10.5560/znb.2011.66c0031

206. Dong, J.; Zhou, Y.; Li, R.; Zhou, W.; Li, L.; Zhu, Y.; Huang, R.; Zhang, K. New nematicidal azaphilones from the aquatic fungus *Pseudohalonectria adversaria* YMF1. 01019. *FEMS Microbiol. Lett.* **2006**, *264*, 65-69. https://doi.org/10.1111/j.1574-6968.2006.00430.x

90. Yang, Z.; Yu, Z.; Lei, L.; Xia, Z.; Shao, L.; Zhang, K.; Li, G. Nematicidal effect of volatiles produced by *Trichoderma* sp. *J. Asia-Pacif. Entomol.* **2012**, *15*, 647-650. https://doi.org/10.1016/j.aspen.2012.08.002

207. Yuan, Y.; Xiang, M.; Xi, P.; Jiang, Z. Screening of nematicidal isolates from the endophytic fungi of *Quisqualis indica* and optimization of culture conditions for the isolate. *Chin. J. Biol. Control* **2010**, *26*, 474. https://doi.org/10.7324/jabb.2017.50101

72. Wang, F.; Guo, Q.; Wang, L.; Ma, Y.; Zhang, T.; Li, R. Nematicidal activities of bacterial volatiles from *Pseudoduganella violaceinigra* G5-3 and *Novosphingobium pokkalii* G8-2 against the pine wood nematode *Bursaphelenchus xylophilus*. *Chiang Mai J. Sci.* **2019**, *46*, 236-246. https://doi.org/10.1163/15685411-00002920

56. Li, L.; Sun, Y.; Chen, F.; Hao, D.; Tan, J. An alkaline protease from *Bacillus cereus* NJSZ-13 can act as a pathogenicity factor in infection of pinewood nematode. *BMC Microbiol.* **2023**, *23*, 10. https://doi.org/10.21203/rs.3.rs-1798582/v1

157. Ponpandian, L.N.; Rim, S.O.; Shanmugam, G.; Jeon, J.; Park, Y.H.; Lee, S.K.; Bae, H. Phylogenetic characterization of bacterial endophytes from four *Pinus* species and their nematicidal activity against the pine wood nematode. *Sci. Rep.* **2019**, *9*, 12457. https://doi.org/10.1038/s41598-019-48745-6

62. Zeng, L.; Jin, H.; Lu, D.; Yang, X.; Pan, L.; Cui, H.; He, X.; Qiu, H.; Qin, B. Isolation and identification of chemical constituents from the bacterium *Bacillus* sp. and their nematicidal activities. *J. Basic Microbiol.* **2015**, *55*, 1239-1244. https://doi.org/10.1002/jobm.201400798

55. Wang, Y.; Mei, L.; Wu, J.; Zhang, L. Detection and characterisation of a *Bacillus thuringiensis* crystal protein with nematicidal activity against the pinewood nematode *Bursaphelenchus xylophilus*. *Biocontrol Sci. Technol.* **2012**, *22*, 1143-1153. https://doi.org/10.1080/09583157.2012.714743

61. Zheng, L.; Li, G.; Wang, X.; Pan, W.; Li, L.; Hua, L.; Liu, F.; Dang, L.; Mo, M.; Zhang, K. Nematicidal endophytic bacteria obtained from plants. *Ann. Microbiol.* **2008**, *58*, 569-572. https://doi.org/10.1007/bf03175559

82. Dong, J.Y.; Song, H.C.; Li, J.H.; Tang, Y.S.; Sun, R.; Wang, L.; Zhou, Y.P.; Wang, L.M.; Shen, K.Z.; Wang, C.R.; et al. Ymf 1029A−E, Preussomerin Analogues from the Fresh-Water-Derived Fungus YMF 1.01029. *J. Nat. Prod.* **2008**, *71*, 952-956. https://doi.org/10.1021/np800034g

208. Proença, D.N.; Francisco, R.; Santos, C.V.; Lopes, A.; Fonseca, L.; Abrantes, I.M.; Morais, P.V. Diversity of bacteria associated with *Bursaphelenchus xylophilus* and other nematodes isolated from *Pinus pinaster* trees with pine wilt disease. *PLoS One* **2010**, *5*, e15191. https://doi.org/10.1371/journal.pone.0015191

209. Liu, Y.; Ponpandian, L.N.; Kim, H.; Jeon, J.; Hwang, B.S.; Lee, S.K.; Park, S.C.; Bae, H. Distribution and diversity of bacterial endophytes from four *Pinus* species and their efficacy as biocontrol agents for devastating pine wood nematodes. *Sci. Rep.* **2019**, *9*, 12461. https://doi.org/10.1038/s41598-019-48739-4

65. Sun, Y.; Wang, C.; Du, G.; Deng, W.; Yang, H.; Li, R.; Xu, Q.; Guo, Q. Two nematicidal compounds from *Lysinimonas* M4 against the pine wood nematode, *Bursaphelenchus xylophilus*. *Forests* **2022**, *13*, 1191. https://doi.org/10.3390/f13081191

88. Dong, J.Y.; Wang, L.; Song, H.C.; Wang, L.M.; Shen, K.Z.; Sun, R.; Li, G.H.; Li, L.; Zhang, K.Q. Ophiocerol, a novel macrocylic neolignan from the aquatic fungus *Ophioceras dolichostomum* YMF1. 00988. *Nat. Prod. Res.* **2010**, *24*, 1004-1012. https://doi.org/10.1080/14786410902854126

59. Kang, M.K.; Kim, M.H.; Liu, M.J.; Jin, C.Z.; Park, S.H.; Lee, J.M.; Kim, J.; Park, D.; Park, H.R.; Kim, Y.H.; et al. Nematicidal activity of teleocidin B4 isolated from *Streptomyces* sp. against pine wood nematode, *Bursaphelenchus xylophilus*. *Pest Manage. Sci.* **2021**, *77*, 1607-1615. https://doi.org/10.21203/rs.3.rs-690115/v1

63. Liu, M.J.; Hwang, B.S.; Jin, C.Z.; Li, W.J.; Park, D.J.; Seo, S.T.; Kim, C.J. Screening, isolation and evaluation of a nematicidal compound from actinomycetes against the pine wood nematode, *Bursaphelenchus xylophilus*. *Pest Manage. Sci.* **2019**, *75*, 1585-1593. https://doi.org/10.1002/ps.5272
210. Chen, C.; Wang, C.; Guo, Q.; Li, L.; Guo, D. Identification and culture condition study of marine actinomycete HT-8 with nematicidal activity against pine wood nematode. *J. Microbiol.* **2016**, *6*, 18. https://doi.org/10.1163/138855410x543517
25. Hayashi, A.; Fujioka, S.; Nukina, M.; Kawano, T.; Shimada, A.; Kimura, Y. Fumiquinones A and B, nematicidal quinones produced by *Aspergillus fumigatus*. *Biosci., Biotechnol., Biochem.* **2007**, *71*, 1697-1702. https://doi.org/10.1271/bbb.70110
211. Zhang, W.; Wu, X.; Wang, Y. Nematicidal activity of bacteria against *Bursaphelenchus xylophilus* and its fermentation and culture characteristics. *Biotechnol. Bull.* **2019**, *35*, 76. https://doi.org/10.1021/acs.jafc.4c13159.s001
212. Xu, H.; Xu, J.; Zhang, L.; Lin, H. Nematicidal activity of *Bacillus thuringiensis* to *Bursaphelenchus xylophilus*. *Chin. J. Biol. Control* **2010**, *26*, 85. https://doi.org/10.1080/09583157.2012.714743
57. Li, D.; Li, Y.; Wang, X.; Zhang, W.; Wen, X.; Liu, Z.; Feng, Y.; Zhang, X. Engineered pine endophytic *Bacillus toyonensis* with nematocidal and colonization abilities for pine wilt disease control. *Front. Microbiol.* **2023**, *14*, 1240984. https://doi.org/10.3389/fmicb.2023.1240984
213. Zhao, Y.; Yuan, Z.; Wang, S.; Wang, H.; Chao, Y.; Sederoff, R.R.; Sederoff, H.; Yan, H.; Pan, J.; Peng, M.; et al. Gene *sdaB* is involved in the Nematocidal activity of *Enterobacter ludwigii* AA4 against the pine wood nematode *Bursaphelenchus xylophilus*. *Front. Microbiol.* **2022**, *13*, 870519. https://doi.org/10.3389/fmicb.2022.870519
54. Li, L.; Tan, J.; Chen, F. *Bacillus pumilus* strain LYMC-3 shows nematicidal activity against *Bursaphelenchus xylophilus* via the production of a guanidine compound. *Biocontrol Sci. Technol.* **2018**, *28*, 1128-1139. https://doi.org/10.1080/09583157.2018.1514587
